# Genome-wide identification of DNA-PKcs-associated RNAs by RIP-Seq

**DOI:** 10.1038/s41392-019-0057-6

**Published:** 2019-07-05

**Authors:** Zhiquan Song, Ying Xie, Zongpei Guo, Yang Han, Hua Guan, Xiaodan Liu, Teng Ma, Ping-kun Zhou

**Affiliations:** 1 Department of Radiation Toxicology and Oncology, Beijing Key Laboratory for Radiobiology, Beijing Institute of Radiation Medicine, 100850 Beijing, People’s Republic of China; 2 Department of Preventive Medicine, Medical School of Hunan Normal University, 410078 Changsha, People’s Republic of China; 3 Institute for Environmental Medicine and Radiation Hygiene, School of Public Health, University of South China, 421001 Hengyang, Hunan Province People’s Republic of China; 4 Department of Cellular and Molecular Biology, Beijing Chest Hospital, Capital Medical University/Beijing Tuberculosis and Thoracic Tumor Research Institute, 101149 Beijing, China

**Keywords:** Molecular medicine, Isolation, separation and purification, Molecular biology

**Dear Editor,**

The DNA-dependent protein kinase catalytic subunit (DNA-PKcs) forms a serine/threonine protein kinase complex with the Ku heterodimer (Ku70/Ku80) and plays an important role in the DNA damage response (DDR) and maintenance of genomic stability through nonhomologous end joining (NHEJ), wherein the Ku heterodimer recognizes and binds broken DNA ends, facilitating the recruitment and activation of DNA-PKcs.^1^ Activated DNA-PKcs phosphorylates and alters the function of factors that mediate NHEJ, including DNA-PKcs itself.^2^ In addition, DDR-independent roles of DNA-PKcs have been demonstrated.^3^ Studies further identified DNA-PKcs as a modulator of cancer-associated pathways distinct from DNA repair, including hypoxia, metabolism, the inflammatory response, and transcriptional regulation.

Although DNA-PKcs’s roles in Double Strand Breaks (DSB) repair and transcriptional regulation are well established, how the activity of DNA-PKcs or the DNA-PK complex is regulated remains mysterious. It has been previously reported that Ku70/Ku80 directly interacts with the telomerase RNA component, hTR^4^ and that hTR stimulates DNA-PK kinase activity, which subsequently phosphorylates hnRNP A1.^5^ The interplay between the DNA-PKcs/DNA-PK complex and RNA functions has emerged as an interesting topic in recent years. Long noncoding RNA *LINP1* acts as a scaffold that links Ku and DNA-PKcs and enables efficient DNA double-strand break repair through nonhomologous end joining (NHEJ).^6^ Given these intriguing examples, here we investigated whether DNA-PKcs may associate with RNA on a genome-wide scale.

To characterize the genome-wide landscape of DNA-PKcs-associated RNAs, we used RNA immunoprecipitation coupled with next-generation sequencing (RIP-seq) to map the DNA-PKcs–RNA interactome in U2OS cells, as depicted in Fig. 1a. Nuclear RNAs immunoprecipitated by an α-DNA-PKcs antibody and control IgG were isolated from U2OS cells exposed to ionizing radiation (0.5 h and 1 h after radiation) and from unexposed cells (0 h) as the control. The α-DNA-PKcs antibody precipitated ~150 ng of RNA from 10^7^ U2OS cells, while precipitation with IgG and precipitation in the no-IR control cells pulled down ~10-fold less RNA (~15 ng), as shown in Fig. 1b. Then, we computationally filtered out adaptors/primer dimers, rRNA, mitochondrial RNA, reads with <18 nt or indeterminate nucleotides, and homopolymer runs in excess of 15 bases. In total, ~1.2 million reads remained after filtering.

**Fig. 1:**
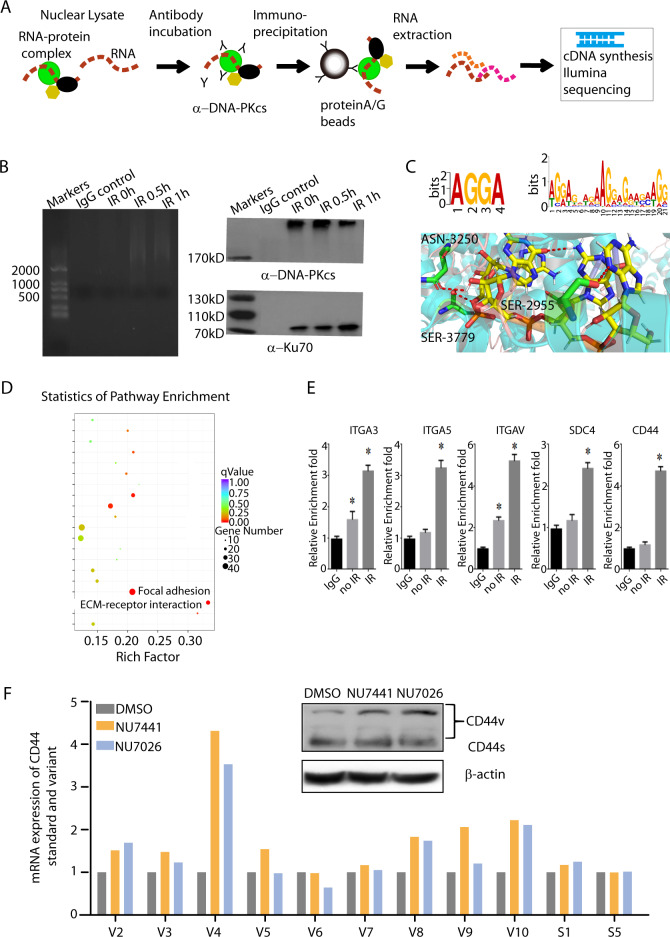
**a** Flow chart of DNA-PKcs RIP-Seq**. b** RNA electrophoresis of the immunoprecipitated RNA by an antibody specific for DNA-PKcs. **c** Motif analysis and docking interaction between DNA-PKcs and RNA-binding sites. **d** KEGG analysis of DNA-PKcs-bound RNAs. **e** RIP-qPCR validation of DNA-PKcs and RNA-binding partners. **f** DNA-PKcs inhibition altered the splicing pattern of CD44 RNA

Then, motif analysis was performed using MEME and DREME software to detect significant motif sequences in the peak sequence. The Tomtom software was used to compare the obtained motif sequence against the known Motif database and annotate it using known motifs. The sequence logo in Fig. 1c shows both the short motif (<8) and long motif (6–30) with the highest binding potential. Motif analysis showed that DNA-PKcs preferentially binds the AGGA sequence, which was in accordance with previous findings (Fig. 1c).^7^ Then, the docking between DNA-PKcs RNA-binding sites deduced from the web server PRIdictor and the RNA motif AGGA was performed on the FRODOCK webserver, and the docking structure was analyzed using PyMOL software (Fig. 1c).

After analysis with a stringent cutoff, ~500 RNAs were precipitated by DNA-PKcs. To categorize the RNAs bound by DNA-PKcs, we performed KEGG analysis. This analysis showed a number of signatures involved in the Focal adhesion and Receptor-ECM interaction pathways (Fig. 1d). Then, the ITGA3, ITGA5, ITGAV, SDC4, and CD44 RNAs were selected for validation of the RIP-Seq results. The RIP-qPCR results showed different fold enrichment values for these five RNAs, which are involved in cell adhesion (Fig. 1e).

Regulation of RNA alternative splicing is a crucial process in RNA-binding proteins function, and aberrant splicing is often associated with various human diseases including cancers;^8^ therefore, to discern how DNA-PKcs modulates bound RNAs, we sought to determine whether DNA-PKcs could affect CD44 alternative splicing. Specific primers to amplify the CD44 standard sequence and variants were designed, and qPCR was performed to examine the expression of different variants after U2OS cells were treated with NU7441 and NU7026, which target DNA-PKcs. The results showed that V4, V9, and V10 increased. Next, western blotting further verified that after DNA-PK inhibition, expression of CD44 variants increased (Fig. 1f).

In summary, our findings strongly support a model wherein the DNA-PKcs protein controls a variety of biological processes, including alternative splicing, through its RNA-binding activity. Further work will elucidate the accessory factors of DNA-PKcs in regulating alternative splicing and how alternative splicing may contribute to the DNA damage response mediated by DNA-PKcs.

## Supplementary Information

Supplementary Information
