# Supplementary material for: Genome-wide identification of DNA-PKcs-associated RNAs by RIP-Seq
Source: Signal Transduct Target Ther. 2019 Jul 5;4:22. doi: 10.1038/s41392-019-0057-6 (PMC6799803; doi:10.1038/s41392-019-0057-6)
Supplement: Supplementary file 1 — Supplementary Information [file 41392_2019_57_MOESM1_ESM.docx]

**Supplementary References:**

Timothy L. Bailey and Charles Elkan (1994)."Fitting a mixture model by expectation maximization to discover motifs in biopolymers", Proceedings of the Second International Conference on Intelligent Systems for Molecular Biology, pp. 28-36, AAAI Press, Menlo Park, California.

Bailey TL (2011). DREME: motif discovery in transcription factor ChIP-seq data. Bioinformatics. 27(12):1653-9.

Gupta S, Stamatoyannopoulos JA, Bailey TL, Noble WS (2007). Quantifying similarity between motifs. Genome Biol. 8(2):R24.

Tuvshinjargal N, Lee W, Park B, Han K (2016). [PRIdictor: Protein-RNA Interaction predictor.](https://www.ncbi.nlm.nih.gov/pubmed/26607710) Biosystems. 139:17-22.

E. Ramírez-Aportela, J.R. López-Blanco, and P. Chacón (2016). FRODOCK 2.0: Fast Protein-Protein docking server. Bioinformatics, 32(15), 2386-2388.
